# Tomographic evaluation of the contralateral ear in patients with severe chronic otitis media

**DOI:** 10.5935/1808-8694.20130085

**Published:** 2015-10-08

**Authors:** Maurício Noschang Lopes da Silva, Jader dos Santos Muller, Fábio André Selaimen, Daniele Sparemberger Oliveira, Letícia Petersen Schmidt Rosito, Sady Selaimen da Costa

**Affiliations:** a MSc. in Surgery (Otologist, Ear Clinic of the Mãe de Deus Health Care Center).; b Specialist in Radiology (Neuroradiologist, Moinhos de Vento Hospital).; c MD (General Practitioner).; d MD (General Practitioner).; e MSc. in Surgery - UFRGS (ENT, Porto Alegre Teaching Hospital; Otologist, Mãe de Deus Health Care Center).; f PhD in Surgery - Ribeirão Preto/USP (Professor of Otorhinolaryngology at the School of Medicine of the UFRGS). Otology of the Mãe de Deus Health Care Center.

**Keywords:** cholesteatoma, otitis media, radiology, tomography

## Abstract

Some studies indicate a bilateral tendency of chronic otitis media. It is believed that the contralateral ear can provide evidences of the route of formation of ear disease in the most affected, be a parameter of Eustachian tube function and predict successful treatment. The CT scan is an excellent test to evaluate the structures of the temporal bone and the changes resulting from otitis media.

**Objective:**

To evaluate Temporal Bone Computed Tomography of patients with chronic otitis media and describe abnormalities in the contralateral ear.

**Method:**

Cross-sectional study. Evaluation of CT scans of 75 patients with chronic otitis media from a tertiary referral hospital in Brazil by a neuroradiologist.

**Results:**

Population was consisted of 50.6% males with a mean age of 36 years. We found 54.7% of changes in contralateral ear clearly associated with chronic otitis media.

**Conclusion:**

The prevalence of radiographic changes in the contralateral ears of patients with chronic otitis media corroborates with clinical, histopathological and functional resources developed by the same group that this disease has a bilateral feature.

## INTRODUCTION

Otitis media is one of the most prevalent diseases in the world. With annual costs estimated at approximately U$ 5 billion in the United States, otitis media is the second leading cause of outpatient visits in the American population younger than 15 years^1^, ^2^, ^3^. According to Sadé et al.^4^, ^5^, chronic otitis media (COM) affects 0.5% to 30% of individuals in any community and over 20 million people are estimated to suffer from this condition worldwide. Harker^6^ found an incidence of cholesteatomas of 6 per 100,000 inhabitants/year in Iowa, United States. For COM, an overall US statistic reported an incidence of 18 cases per 100,000 inhabitants/year, 4.2 associated with the presence of cholesteatoma^7^.

The definition of COM is based on clinical and pathological features. COM is traditionally characterized by the presence perforations, cholesteatoma, recurrent ear discharge, and hearing loss. Histopathologically it is defined by the presence of middle ear inflammation, associated with irreversible tissue damage, regardless of the presence of tympanic membrane perforations^8^.

The disease often evolves in a continuum^1^. Abnormalities that at first caused mild or minimal symptoms, such as simple retractions, for example, can progress to severe changes, such as retraction pockets and destructive cholesteatomas. This continuum model^1^, ^9^, ^10^ explains the development of COM in a progressive manner. According to this theory, effusion, perforations, and cholesteatomas represent different pathological stages of the same disease. The evolution of this continuum can be seen in the contralateral ear (CLE). When tubal dysfunction is the trigger of COM, there is a high probability of impairment of both ears, although in different intensity. Some studies point to a tendency of bilateral involvement in inflammatory pathologies of the middle ear.

Based on this hypothesis, some research has now begun to focus on the contralateral ear of patients affected by COM. Costa et al.^11^ published in this journal the presence of abnormalities in the otoscopic examination of 75% of the CLE of 500 patients with COM; the prevalence of such findings was even higher in the subgroup of patients with cholesteatomas (83%). In a histological study, Rosito et al.^12^ found abnormalities in 91% of the CLE of patients with COM. In a functional study developed by the same research group, audiometric evaluation was performed in 463 patients with COM and showed that about 30% had some degree of hearing impairment in the CLE^13^. In summary, evidence shows that, in patients with COM, the CLE often presents clinical, histopathological and hearing abnormalities. However, there are no radiological studies of the CLE of patients with COM. It is important conclude contralateral ear studies with a tomographic evaluation.

Computed tomography (CT) is an excellent method to visualize bone and pneumatized spaces. It is therefore the best diagnostic method to evaluate the involvement of temporal bone structures resulting from chronic inflammation. There are well-documented findings of COM in CT studies^14^. Modern techniques of helical image acquisition and high-resolution cuts allow the evaluation of smaller structures more accurately.

A comprehensive understanding of the role of the CLE in the evaluation of patients with COM is essential, especially when viewing otitis media from a continuum perspective. We believe that the study of the CLE can provide clues to the pathophysiology of the primary ear disease (ear with full-blown disease), serve as a parameter of tubal function, and contribute to the therapeutic planning.

### Objective

To assess the prevalence of CT abnormalities in the contralateral ear of patients with COM.

## METHOD

The study was carried out with patients seen at the Chronic Otitis Media Outpatient Clinic at Hospital de Clínicas de Porto Alegre (HCPA), Brazil. Patients were referred for CT scans based on clinical judgment alone, independently of participation in this study. Only the most severe cases of COM are referred for CT examination. Among these are patients with persistent effusion, refractory to drug treatment, conductive hearing loss above 30 dB or associated with sensorineural hearing loss, and presence of cholesteatomas. Thus, the study population is a convenience sample of patients with COM, 5 years old or more, who were referred for CT scans, after 2007, based on the criteria described above. We enrolled the first 75 patients who attended the clinic presenting their CT results. Exclusion criteria were previous surgical procedure (except ventilation tubes), known congenital malformations, trauma and other temporal bone pathologies.

A neuroradiologist blinded to clinical information and diagnosis evaluated CT scans. Images were evaluated based on a 38 question protocol focusing on the structures considered to be the most important in the radiological analysis of the temporal bone^15^.

Examinations were performed on a Philips helical CT scanner. Coronal and axial images were obtained with a 0.65 mm slice thickness. Next, based on the patient's clinical presentation, we established which side represented the primary ear and the contralateral ear. The primary ear was defined as the ear presenting more intense symptoms, greater hearing loss or showing more advanced disease in the otoscopic examination^11^, ^12^.

Research procedures and goals were explained to all patients, who also signed a written informed consent. This study was approved by the Institutional Review Board of the *Hospital de Clínicas de Porto Alegre* and registered under the number 10-0569.

### Study design

Cross-sectional (prevalence study).

### Sample size calculation

There are no similar studies published in the literature. In order to estimate a prevalence of 25% of abnormalities, with an absolute margin of error of 10% and a confidence level of 95%, a sample of 72 patients would be required.

### Statistical analysis

Statistical analyses were performed using the software Statistical Package for Social Science (SPSS) 10.0 for Windows. Only prevalence descriptions was performed. There was not statistical tests.

## RESULTS

The main demographic characteristics are described in Table 1.

**Table 1:** Characteristics of the study population.

Demographic characteristics	
Sex	37♀ : 38♂

Age	Mean: 36.4 years SD 19.9 years
	Range: 5-81 years

Contralateral ear	38 left : 37 right

## Study of the contralateral ear

We found a large prevalence of radiological abnormalities in the contralateral ear of patients with COM. The main findings are presented in Table 2.

**Table 2:** Description of the radiological findings in the contralateral middle ear.

Tympanic cavity	
Mesotympanum	

Aeration	54 (72.0%)
Thickened mucosa	12 (16.0%)
Opacification	9 (12.0%)

Hypotympanum	

Aeration	60 (80.0%)
Thickened mucosa	8 (10.7%)
Opacification	7 (9.3%)

Protympanum	

Aeration	61 (81.3%)
Thickened mucosa	11 (14.7%)
Opacification	3 (4.0%)

Malleus	

Normal	68 (90.7%)
Erosion	7 (9.3%)

Incus	

Normal	67 (89.3%)
Erosion	8 (10.7%)

Epitympanum	

Normal	56 (74.7%)
Opacification	18 (24.0%)
Tympanosclerosis	1 (1.3%)

Prussak's space	

Normal	59 (78.7%)
Opacification	16 (21.3%)
Thickened mucosa	-

Within the tympanic cavity, the prevalence of some degree of inflammation was 28%, 20%, 18%, and 25% in the mesotympanum, hypotympanum, protympanum, and epitympanum respectively. Posterior recesses were filled by soft tissue density material in 24% of facial recesses and sinus tympani.

Similar findings were observed in mastoid structures. We found 65.3% well-pneumatized mastoids; 6.7% diploic and 28% sclerotic. Table 3 presents findings for each mastoid compartment.

**Table 3:** Mastoid compartments findings in the contralateral ear.

Mastoid compartment	
Antrum	

Pneumatized	54 (72.0%)
Opacified/poorly pneumatized without trabecular destruction	18 (24.0%)
Opacified/poorly pneumatized with trabecular destruction	3 (4.0%)

Mid mastoid	

Pneumatized	45 (60.0%)
Opacified without trabecular destruction	28 (37.3%)
Opacified with trabecular destruction	2 (2.7%)

Apex	

Pneumatized	34 (45.3%)
Opacified without trabecular destruction	-
Opacified with trabecular destruction	0 (0%)

Some findings are suggestive of a more aggressive COM. Table 4 describes findings for commonly affected structures.

**Table 4:** Radiological signs of complications in the temporal bone of the contralateral ear.

Chaussé spur	
Normal	68 (90.7%)
Eroded	7 (9.3%)

Lateral semicircular canal	

Normal	73 (97.3%)
Fistula	2 (2.7%)

Tympanic tegmen	

Normal	74 (98.7%)
Eroded	1 (1.3%)

Cortical layer of posterior fossa	

Normal	75 (100%)
Eroded	-

Facial nerve canal [tympanic segment]	

Normal	70 (93.3%)
Eroded/Dehiscence	3 (4.0%)
Not visualized	2 (2.7%)

Facial nerve canal [mastoid segment]	

Normal	72 (96.0%)
Eroded	2 (2.7%)
Not visualized	1 (1.3%)

It is possible that some of the abnormalities found at CT were not attributable to COM. In order to summarize findings inarguably secondary to COM, we present the prevalence of some variables clearly associated with the disease: middle ear opacification, epitympanum opacification, Chaussé spur erosion, and mastoid sclerosis or opacification. The prevalence of patients showing at least one of these abnormalities was 54.7%. Out of these, 6.7% were patients with signs of some form of complication, such as cortical erosion of the mastoid, tegmen erosion, or fistula of the lateral semicircular canal.

## DISCUSSION

We believe that a careful study of the contralateral ear can significantly improve our understanding of the pathogenesis of COM^16^, ^17^. Moreover, a thorough analysis of both ears can help to establish three essential aspects in the development of COM: etiology, current status, and disease evolution, both in terms of speed and direction. By valuing and assessing the CLE, we have the opportunity to see “now” what happened to the primary ear “yesterday”.

**Figure 1:**
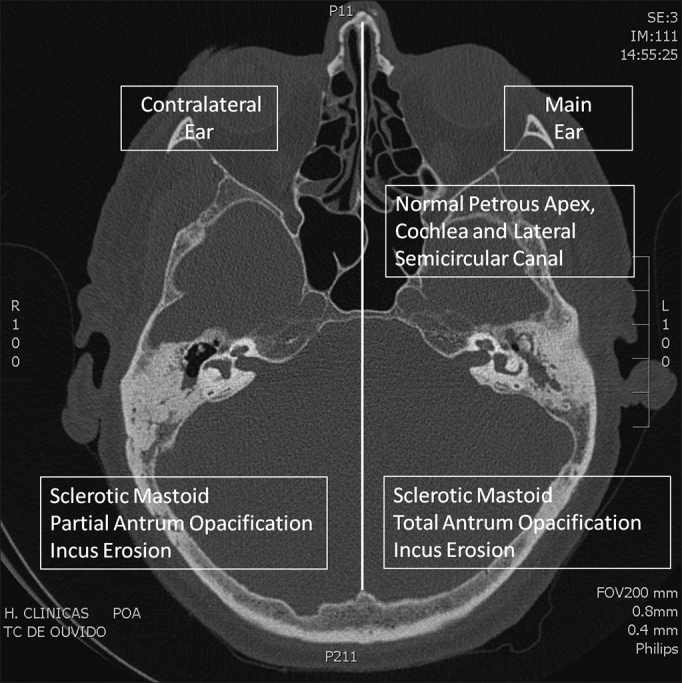
Axial CT Scan. Comparison of abnormalities in the main (left) and contralateral (right) ear.

**Figure 2:**
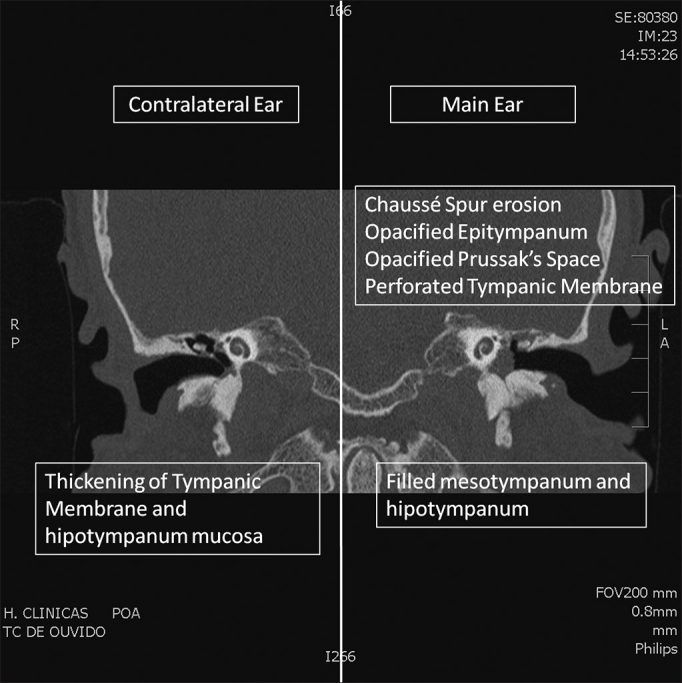
Coronal CT Scan. Comparison of abnormalities in the main (left) and contralateral (right) ear.

The large number of radiological signs of COM found in the contralateral ears confirms the importance of studying both ears (Figures 1 and 2). The prevalence of opacification or mucosal thickening in the tympanic cavity was approximately 20%. Similar percentages were found when we evaluated only the region of the protympanum, and it should be noted that obliterations in this area may be responsible for tubal dysfunction, reduced transmucosal gas exchange, and development of otitis media^9^. However, it is important to remember that unlike otoscopic^6^ and audiometric^8^ studies, in which all patients with COM are evaluated, our study population involved patients with chronic otitis media who had an indication to perform CT. Clearly, this translates into patients with more severe disease. Patients with cholesteatomas, retractions with major hearing loss, and refractory effusions were included.

It is likely that our study population presents tomographically more affected CLE than a typical sample of patients with dry central perforations. However, we do not believe it would be appropriate to expose patients to radiation from a CT scan without clinical indication. Even though we recognize that patients enrolled in this study may overestimate the prevalence of radiological abnormalities, the existence of such abnormalities in the contralateral ear is indisputable and reinforces findings from previous studies. Nevertheless, we emphasize that the external validity of this research is limited to similar populations, mainly, patients with cholesteatomas.

The mastoid structures also showed a high prevalence of abnormalities (35%). This may have some implication in the pathophysiology of otitis media, as the data suggest the occurrence of some disturbance in the development of the mastoid that affects both ears considerably. It is important to highlight that although some clinical and radiological findings are due to active, current disease (for example, tympanic membrane retractions seen at otoscopic examination or mucosal thickening in the middle ear seen on CT), most of the changes described in CT are the result of the entire disease process.

The classical model is that of a child who develops COM, with impairment of middle ear ventilation and, consequently, reduced mastoid pneumatization. Even if a therapeutic or spontaneous resolution occurs, the mastoid will remain radiologically abnormal in adulthood. Bony erosions or ossicular disorders that took place in the past will still be observed on the CT. This concept has two implications: while there may be an increased prevalence of radiographic changes compared to patients who are currently effectively ill, the exam also allows for the identification of previous pathological processes that occurred in the middle ear.

## CONCLUSION

We conclude that the contralateral ear presents a high prevalence of CT abnormalities in patients with severe COM.
